# Spatio-temporal Patterns and Landscape-Associated Risk of Buruli Ulcer in Akonolinga, Cameroon

**DOI:** 10.1371/journal.pntd.0003123

**Published:** 2014-09-04

**Authors:** Jordi Landier, Jean Gaudart, Kevin Carolan, Danny Lo Seen, Jean-François Guégan, Sara Eyangoh, Arnaud Fontanet, Gaëtan Texier

**Affiliations:** 1 Institut Pasteur, Unité de Recherche et d'expertise en Epidémiologie des Maladies Emergentes, Paris, France; 2 Service d'Epidémiologie et de Santé Publique, Centre Pasteur du Cameroun, Réseau International des Instituts Pasteur, Yaoundé, Cameroon; 3 Aix-Marseille Université, UMR912 SESSTIM (INSERM - IRD - AMU), Marseille, France; 4 UMR MIVEGEC 5290 CNRS - IRD - Université de Montpellier I - Université de Montpellier II, Montpellier, France; 5 UMR TETIS, CIRAD, Montpellier, France; 6 Service de Mycobactériologie, Centre Pasteur du Cameroun, Réseau International des Instituts Pasteur, Yaoundé, Cameroon; 7 Chaire Santé et Développement, Conservatoire National des Arts et Métiers, Paris, France; Institut Pasteur, France

## Abstract

**Background:**

Buruli ulcer (BU) is an extensively damaging skin infection caused by *Mycobacterium ulcerans*, whose transmission mode is still unknown. The focal distribution of BU and the absence of interpersonal transmission suggest a major role of environmental factors, which remain unidentified. This study provides the first description of the spatio-temporal variations of BU in an endemic African region, in Akonolinga, Cameroon. We quantify landscape-associated risk of BU, and reveal local patterns of endemicity.

**Methodology/Principal Findings:**

From January 2002 to May 2012, 787 new BU cases were recorded in 154 villages of the district of Akonolinga. Incidence per village ranged from 0 (n = 59 villages) to 10.4 cases/1000 person.years (py); median incidence was 0.4 cases/1,000py. Villages neighbouring the Nyong River flood plain near Akonolinga town were identified as the highest risk zone using the SPODT algorithm. We found a decreasing risk with increasing distance to the Nyong and identified 4 time phases with changes in spatial distribution. We classified the villages into 8 groups according to landscape characteristics using principal component analysis and hierarchical clustering. We estimated the incidence ratio (IR) associated with each landscape using a generalised linear model. BU risk was highest in landscapes with abundant wetlands, especially cultivated ones (IR = 15.7, 95% confidence interval [95%CI] = 15.7[4.2–59.2]), and lowest in reference landscape where primary and secondary forest cover was abundant. In intermediate-risk landscapes, risk decreased with agriculture pressure (from IR[95%CI] = 7.9[2.2–28.8] to 2.0[0.6–6.6]). We identified landscapes where endemicity was stable and landscapes where incidence increased with time.

**Conclusion/Significance:**

Our study on the largest series of BU cases recorded in a single endemic region illustrates the local evolution of BU and identifies the Nyong River as the major driver of BU incidence. Local differences along the river are explained by wetland abundance and human modification of the environment.

## Author Summary

Buruli ulcer (BU) remains a mysterious disease without efficient prevention since the mode of transmission of its agent, *Mycobacterium ulcerans*, is still unknown. The disease is highly localised within countries and even at the village scale within endemic regions, but environmental factors explaining this focal distribution have not been described yet. In this article, we rely on a large series of cases originating from Akonolinga region, Centre region, Cameroon, and recorded at the BU treatment center of the hospital of Akonolinga. The series of 787 patients over 10 years allows us to describe the distribution of BU incidence in the region and its changes over time and space. We identify the Nyong River as a major risk factor, and identify environmental factors along the river that further increase the risk of BU, such as the high proportion of swamps, the degradation of forests and cultivation of lands by human populations. These results will help to locate where the transmission is most likely to happen, and provide useful elements for targeting case search, prevention actions and future research on *M. ulcerans* transmission.

## Introduction

Buruli ulcer (BU) disease is an extensively damaging skin infection caused by *Mycobacterium ulcerans* (MU), a pathogen distantly related to *Mycobacterium tuberculosis* and *M. leprae* [1], [2]. BU presents as a necrotising infection of the skin, causing debility and crippling deformity if left untreated. Initially described in Uganda and Australia [3], BU has been reported in 33 countries and mainly prevalent in tropical regions. In 2011, 4,009 cases were reported to the World Health Organization (WHO) by 14 countries [4]. The majority of BU cases (96%) originated from countries around the gulf of Guinea and Cameroon reported 256 cases.

Means of preventing the infection are still lacking as the mode of transmission of MU to humans remains unknown [3], [5]. It is unclear where the microbe resides in the environment: genomics data suggest recent adaptation to a new environmental niche and specialisation to a given host [6], [7] while environmental detection studies across a wide diversity of samples (insect and vertebrate fauna, water filtrates, vegetal debris) seem to indicate that the microbe could occupy a wide diversity of environments [5], [8]–[10]. How the microbe reaches human skin is also highly debated: a role of insects as vectors has been hypothesised [9], [11], but remains controversial [5].

In this context of unknown transmission mechanisms and unknown environmental location of the pathogen, the study of spatial and temporal variations of BU incidence could identify where and when MU transmission events are most likely to occur and provide insights towards understanding the elusive epidemiology of BU [12], [13]. BU incidence has been described as highly focal in countries such as Uganda, Benin, Côte d'Ivoire and Ghana, where endemic regions are usually well defined. In Cameroon, three foci are identified, the Nyong valley in Ayos and Akonolinga (Centre region), Bankim (Adamaoua region) and Mbongue (South-West region), but sporadic cases originate from various places across the country, suggesting that the description of BU endemic regions is incomplete [14]. Descriptive maps of BU incidence or prevalence were established in Uganda as early as the 1970s [15], [16]. Since that period, several maps of prevalence or incidence rate have been established in endemic regions from Cameroon [17]–[19], Democratic Republic of Congo [20], Benin [21], Côte d'Ivoire [22], [23] and Ghana [24], [25]. These maps showed that the distribution of BU is highly focal at country scale, and also within endemic regions [16], [17], [19].

Several studies have shed light on spatial patterns of BU distribution and environments associated to BU at the national scale [21], [23], [26] or at the regional scale [27], [28]. The environmental factors associated to BU prevalence or incidence were: low elevation in Benin [21], [27] and South Australia [28], high percentage of forest cover and low percentage of urban cover in Côte d'Ivoire [23], Benin [21] and South Australia [28]. In Benin, the standard deviation of wetness index, an indicator of areas with contrasted topographic features, was associated with a higher risk of BU [21]. In Côte d'Ivoire, irrigated rice producing areas and the proximity of remnant rainforest patches were associated with a higher risk of BU [23]. These analyses contributed to identifying the characteristics of the regions at risk within countries, and a model from Benin could even be used to predict where these regions would be in neighbouring Ghana [26]. However, they provided little insight on the local determinants of BU prevalence within endemic regions, where endemic and non-endemic villages can be very close. The spatial resolution was probably too low in these studies to distinguish local variations in prevalence or to provide sufficient contrasts in the descriptions of the environment [26], [28].

In this article, we present the first analysis of BU incidence patterns at the village level in an African endemic region, the Akonolinga health district, Centre region, Cameroon. This analysis is based on one of the largest series of cases available to date and on a fine scale characterisation of the environment. The objectives of this study were: 1) to describe the local spatial patterns and spatio-temporal variations of BU incidence; 2) to characterise and quantify the environmental factors associated with high BU incidence in Akonolinga district.

## Materials and Methods

### Setting

This study was performed in Akonolinga health district, located 100 km east from Yaoundé in the Centre Province of Cameroon. The predominant environment is tropical forest and the district is crossed by the Nyong River which flows from east to west. BU was first described in this area in 1977 [29]. BU prevalence in the district was described in 2001 [17] and 2007 [18]. Risk factors for BU have also been investigated in a case control study in 2006 and several individual risk factors related to the environment were identified, such as having activities in the Nyong River, or having forest or a cacao plantation close to the habitation [30]. The present study was conducted based on BU cases recorded for Disease Surveillance activities from January 2002 (start of the treatment intervention) to May 2012.

### BU case data

This study relied on the analysis of the registry [29] of BU patients included in the BU management intervention at Akonolinga District Hospital, for which the Centre Pasteur du Cameroun (CPC) performed biological confirmation of MU infection diagnosis as the National Reference Laboratory. All new patients treated for BU in Akonolinga after clinical diagnosis, and with a documented place of residence in Akonolinga district, were included in the analysis. Patients without a documented village of residence or with an unidentified village of residence were excluded. These data were collected routinely at Akonolinga District Hospital and at Centre Pasteur du Cameroun as part of the BU Disease Surveillance system of the National Control Program.

A clinical case was defined as a patient with a clinical diagnosis of BU, made at the Akonolinga District Hospital, by trained specialized health practitioners in charge of the BU treatment. A confirmed case was defined as a clinical case with a positive result for at least one of the two biological confirmation methods, microscopy [31] or PCR [32] which are performed routinely by the CPC as the National Reference Laboratory according to WHO recommendation [31]. Laboratory confirmation could not always be obtained, however clinical diagnosis was shown to be very reliable in endemic regions [33].

### Ethics statement

This study used anonymised case data, aggregated by village and by month, which were collected by the Service de Mycobactériologie of the Centre Pasteur du Cameroun as part of the surveillance activity of the National Reference Laboratory for BU in Cameroon, within the National BU Control Program. In this study, no intervention was performed (either diagnostic or therapeutic) and we only relied on a retrospective collection of anonymous cases authorized by the Cameroonian Ministry of Health.

### Population data

Population data for Akonolinga health district villages were obtained from the national population census bureau (BUCREP). These population data were a 2010 projection based on the detailed results of the 2005 census. Population settlements in the Centre region are typically hamlets relatively close to each other which form a village (“chefferie”) under the administrative authority of a traditional chief [34], [35]. Since cases were reported at the village level, we aggregated the population of hamlets at village level.

For the towns of Akonolinga and Endom, the urban neighbourhoods were aggregated.

### Administrative data

In accordance with Cameroonian laws (decree 77/245), a village was defined as the collection of all hamlets under the jurisdiction of the same traditional chief and was represented on the maps as the surface encompassing all hamlets. Hamlets had been either geolocated using a GPS during previous fieldwork [30], [36] or identified on a 1/2,00 000 scale map (Institut National de Cartographie, Yaoundé, sheets of Yaoundé, Nanga Eboko and Akonolinga).

Topographical and environmental data were extracted using a circular 5 km-radius buffer around the village centroid. This value of 5 km was chosen based on a socio-anthropologic evaluation done in the region (described in [37]) and it approximated the distance that could easily be walked by inhabitants for their daily activities: fishing, farming, going to school. Furthermore, each hamlet was located within the 5-km-radius buffer of its village.

### Topographical data

Topography has been shown to be a major driver in most studies [21], [23], [27]. A digital elevation model was used to obtain elevation data (Shuttle Radar Topography Mission, available from the U.S. Geological Survey). A map of 90 m-topographic wetness index (TWI), an indicator of zones where water tends to accumulate due to abundant runoff from the surrounding area and a low slope, was obtained from Africa Soil Information Service (http://www.africasoils.net). TWI was categorised around the value 18, since following fieldwork, TWI>18 corresponded best to the bottoms of valleys which were most likely to represent wetlands. The percentage of each buffer area within this class was used as an indicator of the abundance of wetlands. Data on the distribution of rivers and roads were obtained from IFORA project and Institut National de Cartographie du Cameroun.

### Environmental data

First, we used aggregated measures to quantify vegetation cover in each buffer. We used a vegetation index calculated from remote sensing multispectral data measured by the MODIS satellite. The Enhanced Vegetation Index (EVI) is available from U.S. Geological Survey as a monthly image with 1 km^2^ resolution averaging measurements performed with a 16-day period (30-Day L3 Global 1 km product - MOD13A3). EVI was used as a measurement of overall forest cover: it is directly related to photosynthetic activity and biomass and was developed specifically for high biomass areas such as tropical forests [38]. Using images from December 2001 to December 2011, we calculated the mean EVI during the dry season for each village buffer to approximate vegetation cover. During the dry season, contrast was expected to be maximal between herbaceous or cultivated zones, where annual plants require rain for their growth, and forest where perennial vegetation relying on deep soil water, would still present a high photosynthetic activity [39]. Deforestation was one of the major human-driven changes that we expected in this area. We calculated the mean EVI over months December to February at the beginning and end of the study period (dry seasons 2001–2002 and 2011–12). The difference between the two values was included as a crude proxy for quantitative vegetation change over the study period.

Second, we characterised the environment in more detail (forest type, cultivated areas…) using two distinct Land-use/Land-cover (LULC) datasets.

The first dataset was a classification constructed using two Landsat images from February and March 2001 which were selected for low cloud content. Initial exploratory maps were classified using multi-spectral decision trees in the software ENVI, version 4.8 (Exelis Visual Information Solutions, Boulder, Colorado). Following ground truthing of these initial maps in November 2012, they were refined using object orientated image analysis in the software eCognition (eCognition Developer version 8.9.1, Trimble Geospatial Imaging, Munich, Germany). This resulted in regions classified as Urban, Road, Forest, Crop, Flood plain or Swamp categories.

The second dataset was a map of forest types established in 2002 and obtained from the Forest Atlas of Cameroon [40]. The study area presented 9 classes of vegetation: primary forest (dense humid evergreen or with raffia trees), secondary forest (young or adult, cultivated or not), forested wetlands/swamps, wetlands, and savannah. Secondary forest represents forest growing after being cleared (completely or partially). Two categories are distinguished according to the time elapsed since clearing. Young secondary forest corresponds to the first 5 to 20 years after clearing. It hosts mainly plant species that grow rapidly and in the light. With time, the number and variety of plant species increase, the canopy closes and the forest becomes adult secondary forest, characterised by a high biodiversity. In Akonolinga region, clearing resulted mainly from familial agriculture. The forest category indicated the intensity of human pressure on the environment.

The first dataset was used mainly for urban, agricultural, and wetland land-cover characterisation, which were combined with detailed forest data from the second dataset. New classes or new attributions were derived, such as “cultivated wetlands” corresponding to areas listed as cultivated in one dataset and swamp or swamp forest in the other.

### Statistical analyses

All analyses were performed using R software version 3.0.2 (R Development Core Team, R Foundation for Statistical Computing, Vienna, Austria), including packages DCluster, SPODT, FactoMineR, bcp; and the software ArcGIS version 10.0 (ESRI Inc. Redlands, CA), including the extension Spatial analyst. Graphics were drawn using the ggplot2 R-package and maps were drawn using ArcGIS.

#### Incidence rate calculation and mapping

In order to analyse the distribution of cases in Akonolinga district, a map of the mean monthly incidence rate of BU per village was drawn for each phase and for the cumulative series. Mean incidence rate was expressed in cases per 1,000 person.years (cases/1,000py) and allowed comparisons between villages with different population sizes and different exposure times. To be represented on the maps, incidence rate were discretised using rounded values of the classes obtained by the Jenks method in ArcGIS, which enabled maximization of contrasts.

#### Analysis of spatial clustering

To address the question of whether cases occurred at random in the district or according to a given spatial pattern, we performed several statistical analyses. First, a general statistic of global aggregation, Moran's Index (I), was calculated to assess spatial autocorrelation [41]. Statistical significance was calculated using bootstrap methods. Then, we evaluated the relative risk of BU over the Akonolinga region using the Spatial Oblique Decision Tree algorithm (R package SPODT). This method was used to identify homogeneous risk areas on the time-aggregated series and to quantify the risk associated with each zone. This method is adapted from classification and regression tree techniques and uses straight lines to split the study area in groups of villages as homogenous as possible regarding incidence rate [42]. It identifies clusters without any shape assumption, and is less biased by edge effects. It also provides risk estimates in all areas. Statistical significance was calculated using Monte-Carlo inference. We estimated the relative risk for each zone delimited by SPODT, by calculating an odds-ratio and its 95% significance interval.

#### Spatio-temporal analysis

Based on the spatial analysis, we analysed the incidence distribution over time and space using a “heat-map”, displaying mean monthly incidence for each quarter and for each village after ordering them according to their distance to the Nyong River. We identified several “phases” in the time-series defined as periods of time presenting heterogeneous spatial patterns of incidence. These phases were confirmed using Bayesian change-point detection methods [43] to have a high probability of representing a change in the time series. Maps of incidence were drawn.

#### Classification of villages into landscape groups

We performed a principal component analysis (PCA) on the environmental data extracted for each village on a 5 km-radius buffers (see Supplementary Figure S2 legend in Text S1 for details). This step allowed grouping variables from the different categories, removing colinearity and selecting the most relevant variables for describing the environment in the Akonolinga region. Homogeneous groups of villages with similar landscape environments were built by classifying the villages according to the PCA results using agglomerative hierarchical clustering with a Euclidean distance metric (unsupervised classification).

#### Estimation of landscape-associated risk

In order to estimate the risk associated to each landscape, a generalised linear model (GLM) was built. A binomial negative regression model was preferred, since it was more adapted to this series with count numbers, as in previous BU studies [21]. Categorical variables were included in the model: landscape profile as a single variable with one modality per group, and distance to the Nyong River in 4 categories, ≤5 km, >5–10 km, >10–20 km, >20 km, according to the activity range of populations. The model allowed estimation of an incidence ratio (IR) associated to each class. Interaction between landscape profile and distance to the Nyong River was investigated by splitting the landscape classes in groups of distance when all the villages of one landscape were not included in the same distance class. When it was found significant for one landscape, two subsets landscape were created for the final analysis, distance to Nyong ≤10 km and >10 km based on the distance where Nyong influence was significant.

Univariate and multivariate models were assessed for parsimony using Akaike information criterion (AIC). Fitting was assessed by the percentage of deviance explained.

To assess model performance at representing the spatial variations of BU incidence we mapped the model residuals and explored their distribution using Moran's I statistic. We expected that no autocorrelation would remain if the model accurately captured the spatial pattern of incidence resulting from the different landscapes.

#### Temporal evolution of BU incidence in each landscape

We drew the cumulative incidence graphs over time for each group of villages from the same landscape to examine the local temporal variations of BU. The cumulative incidence over time was fitted with a linear model. When a linear fit was appropriate and indicated constant incidence, average incidence in the landscape was calculated for the period 2002–2012. Exponential fit was also tested by fitting a curvilinear model to the logarithm of cumulative incidence.

## Results

### BU case data

From January 2002 to May 2012, 915 patients originating from Akonolinga health district were diagnosed and treated free of charge at the Akonolinga district hospital by the BU management intervention. Out of these patients, 853 were new cases and among those, 787 cases had a documented place of residence in one of the 154 villages of Akonolinga district included in the analysis. The total population of the study villages was 60,188 inhabitants and the study area had a surface of 3,685 km^2^. The north part of the district, 16 villages totalling 10 cases and 2,750 inhabitants, was excluded because the area was only documented in the forest LULC database.

Among the 787 clinical BU cases in the database, 513 (65%) had received a laboratory-confirmed MU infection diagnosis (396 by PCR and/or microscopy, 117 by microscopy only). All 787 clinical BU cases were included in the analysis.

### BU incidence rate and spatial distribution in Akonolinga district

**Figure 1:**
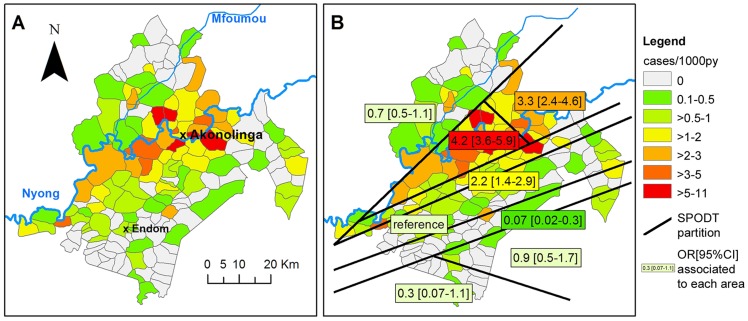
Identification of the Nyong as a major risk factor for BU incidence in Akonolinga 2002–2012 (spatial analysis on time-aggregated incidence rate of BU in Akonolinga). A: Incidence rate per village (cases/1,000py). B: Decreasing risk of BU with increasing distance to the Nyong River. Homogenous risk areas of Akonolinga district were identified using the SPODT algorithm. Associated odds-ratio and 95% CI are provided.

Global BU incidence rate in the study area was 1.25 cases/1,000py over a time period of 10 years and 5 months. Incidence per village ranged from 0, in 59 villages, to 10.4 cases/1,000py, and median incidence was 0.4 cases/1,000py (Interquartile Range = [0–1.1]). A map of cumulative incidence rate over the time-aggregated series is presented in Figure 1A. Cumulative incidence appeared to be highest in villages close to the Nyong River, east of Akonolinga town. There was a significant global aggregation of cases (Moran's I = 0.349, p<10^−6^). The SPODT algorithm identified that the highest risk zone was centered on the Nyong River upstream of Akonolinga (Figure 1B). A decreasing risk gradient with increasing distance to the Nyong was identified, and the highest risk zone had 67 times higher risk of BU than the lowest risk zone.

### Spatio-temporal variations of BU incidence in Akonolinga district, 2002–2012

**Figure 2:**
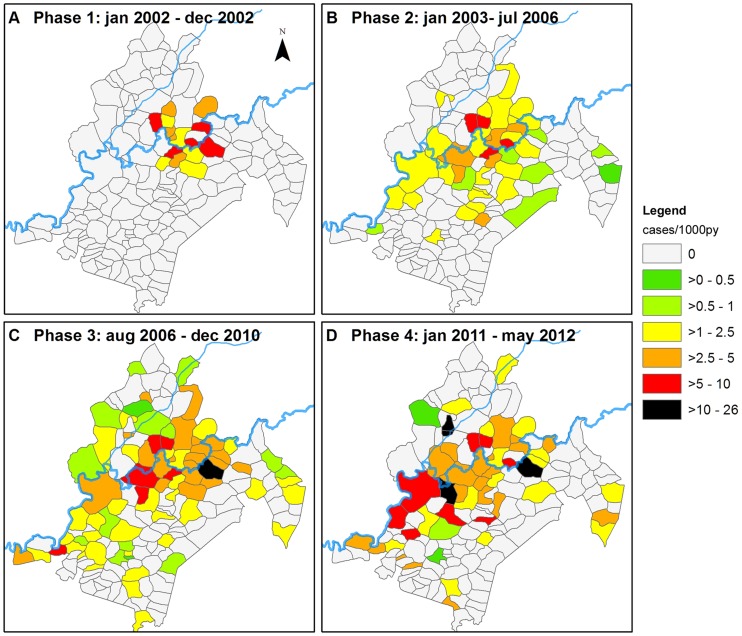
Maps of spatio-temporal variations of BU incidence in Akonolinga district. A–D: Incidence rate maps for the periods, phases 1 to 4, identified in the time-series (cases/1,000py).

The role of the Nyong River as a high risk area, and the decreasing risk gradient away from the river, led us to investigate the temporal variations of BU incidence per village according to their distance to the Nyong (Figure S1 in Supplementary Text S1). By change-point analysis process, we identified four phases corresponding to changes in the disease spatial distribution (Figure 2). In the first phase, corresponding to year 2002, the debuting BU treatment program only recruited cases from Akonolinga town and the neighbouring villages (Phase 1, Figure 2A). In the following phases, recruitment was on the entire district. From being centred on Akonolinga town from 2002 to 2006 (Phase2, Figure 2B), the high incidence area appeared to move, first eastward upstream the Nyong in the area of Abem (Phase 3, Figure 2C), then downstream along the Nyong, on the southern part of the river and on the Mfoumou, a tributary of the Nyong (Phase 4, Figure 2D).

### Analysis of local environmental risk-factors for BU in Akonolinga district

#### Environment classification in landscape groups

The unsupervised classification of villages distinguished 7 landscape groups, organised on two main gradients (Table 1 and details in Supplementary Text S1, Figure S2). First, a clear separation was observed between villages with abundant forest cover compared to those where it was greatly reduced, as indicated by EVI values. This separation allowed the definition of a first gradient of increasing human alteration of landscape, based on abundant urban and agricultural land-use, and low proportion of forest cover. Landscape “Urban Nyong” and landscape “Rural Nyong” were characterised by a low forest cover and abundant areas dedicated to agriculture, as well as a high proportion of wetlands. A second gradient separated the villages according to the forest maturity (primary, secondary adult, secondary young) and the proportion of which was mosaicked with cultures. Landscapes “Forest 1” and “Forest 2” had the most abundant forest cover and were generally at a higher altitude with lower proportion of wetlands. “Forest 1” included remnants of dense humid evergreen primary forest, which marks the persistence of undisturbed ecosystems. “Forest 2” included a high proportion of secondary adult forest cover, a fraction of which was cultivated. Landscapes “Cultivated forest” and “Young forest” included intermediate features between these two groups, where young secondary forest, cultivated or not, dominated, indicating a more intense agricultural pressure. Both also presented abundant proportion of wetlands. Finally, landscape “Savannah” corresponded to 2 villages located in a specific area of savannah within the forest. Changes in forest cover, approximated by EVI difference between dry season 2001–02 and 2011–2012 were highest in landscapes “Forest 2” and “Young forest”.

**Table 1:** Selected environment characteristics of landscape groups defined in Akonolinga district.

Landscape	Major features*	Mean EVI in December*	EVI decrease 2001–12*	Area with WI>18*	Forested Wetland*	Cultivated Wetland*	N villages	Total Population	Main watershed
**Savannah**	Savannah (81%)	0,367	0,049	6%	0	0	2	538	Nyong
**Urban Nyong**	Young cultivated secondary forest (29%), Urban (5%), and cultivated land (9%)	0,381	0,047	37%	7%	8%	7	17813	Nyong
**Rural Nyong**	Young cultivated secondary forest (25%)	0,392	0,062	20%	5%	3%	20	6656	Nyong
**Cultivated Forest <10 km Nyong**	Young cultivated secondary forest (37%)	0,417	0,039	6%	10%	3%	16	5015	Nyong
**Cultivated Forest >10 km Nyong**	Young cultivated secondary forest (39%)	0,416	0,026	7%	10%	2%	31	8529	Dja
**Young Forest**	Young secondary forest (38%)	0,417	0,074	5%	9%	+	12	3069	Nyong
**Forest 2**	Adult secondary forest (50%)	0,415	0,076	5%	0	+	58	16729	Nyong
**Forest 1**	Adult secondary forest (48%) and Primary forest (18%)	0,420	0,068	4%	+	+	8	1839	Dja

*median value for each landscape group; + present, <1%.

#### Estimation of landscape-associated risk

The use of a generalised linear model allowed estimation of BU incidence ratio (IR) from January 2002 to May 2012. Univariate analysis is presented in Table 2. In the landscape model, the highest risk zones corresponded to landscapes “Urban Nyong” and “Rural Nyong” compared to landscape “Forest 1”. “Young forest” presented an intermediate risk and all other landscapes did not significantly differ from “Forest 1”. In the Nyong River distance model, risk decreased with increasing distance to the river with a dose-response relationship.

**Table 2:** Univariate analysis.

		IRR^1^	[95%CI]^2^	p-value
**Landscape group**	Urban Nyong	15.8	4.05–61.32	<0.001
	Rural Nyong	12.6	3.6–44.0	<0.001
	Savannah	5.4	0.8–36.4	0.081
	Cultivated Forest	2.8	0.8–9.5	0.106
	Young forest	7.9	2.1–29.6	0.002
	Forest 2	2.0	0.6–6.7	0.287
	Forest 1	1	reference	
**Distance to the Nyong**	≤5 km	7.5	4.3–13.5	<0.001
	>5 and ≤10 km	2.3	1.2–4.3	0.01
	>10 and ≤20 km	1.2	0.7–2.2	0.528
	>20 km	1	reference	

Incidence rate ratios estimated for the landscape groups and the distance to the Nyong River in 154 villages of Akonolinga district, Cameroon, 2002–2012.

1 IRR: Incidence Rate ratio.

2 [95%CI]: 95% confidence interval.

**Figure 3:**
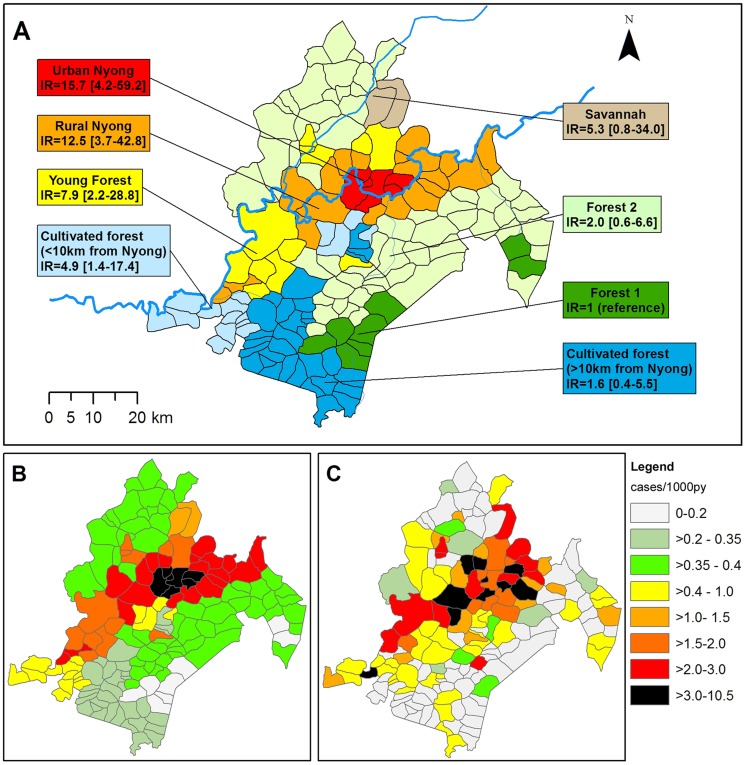
Landscape-associated risk of BU in Akonolinga district, 2002–2012. A: Classification of Akonolinga area villages according to landscape group and associated BU incidence ratio with 95% confidence interval. B: Predicted cumulative incidence for each village of the district according to the landscape model (cases/1,000py). C: Observed cumulative incidence rate for each village of the district (cases/1,000py).

For multivariate analysis, we combined Nyong distance and landscape and split landscape “Cultivated forest” according to the location of villages within or beyond the influence range of the Nyong, i.e. “Cultivated forest, ≤10 km from Nyong” and “Cultivated forest, >10 km from Nyong”. All other landscapes were located within a single Nyong distance class or did not present significant differences in IR between the two distance classes. The resulting model (Table 3 and figure 3A) indicated that “Urban Nyong” and “Rural Nyong” had the highest risk, respectively IR = 15.7 (95%CI = [4.2–59.2]) and IR = 12.5 (95%CI = [3.7–42.8]) compared to landscape “Forest 1”. Landscapes “Young forest” and “Cultivated forest ≤10 km from Nyong” had intermediate risk, respectively IR = 7.9 (95%CI = [2.2–28.8]) and IR = 4.9 (95%CI = [1.4–17.4]). Finally, risk for landscapes “Forest 2”, “Cultivated forest >10 km from Nyong” and “Savannah” did not significantly differ from the “Forest 1” landscape. This model explained 41% of the variance between the villages. We performed a further analysis on the model residuals, and found that their spatial distribution presented no remaining spatial autocorrelation (Moran's I = 0.021, p = 0.65). This indicated that our model was able to capture most of the spatial pattern between the villages. Predicted incidence rate and actual cumulative incidence rate maps are presented in Figure 3B and 3C.

**Table 3:** Incidence rate ratios estimated for the landscape groups combined with Nyong River distance in 154 villages of Akonolinga health district, Cameroon, 2002–2012.

Landscape group	IRR^1^	[95%CI]^2^	p-value
Urban Nyong	15.7	4.2–59.4	<0.001
Rural Nyong	12.5	3.7–42.9	<0.001
Savannah	5.4	0.8–34.2	0.077
Cultivated forest; ≤10 km to Nyong	4.9	1.4–17.4	0.014
Cultivated forest; >10 km to Nyong	1.6	0.4–5.5	0.499
Young forest	7.9	2.2–28.9	0.002
Forest 2	2.0	0.6–6.6	0.277
Forest 1	1	reference	

Deviance explained: 41%; Akaike Information Criterion: 578.4.

1 IRR: Incidence Rate ratio.

2 [95%CI]: 95% confidence interval.

#### Temporal variations of BU incidence in each landscape

We studied the series of monthly incident cases for each landscape in order to characterise the temporal variations of BU incidence within the different landscapes (Supplementary Text S1, Figure S3). Landscape “Forest 1” presented only 4 cases during the study period (incidence of 0.2 cases/1,000py) and “Savannah” only 3 cases (0.5 cases/1,000py). Landscapes “Urban Nyong”, “Rural Nyong”, and “Forest 2” presented stable incidence rates over the study period, averaging respectively 2.1, 2.4 and 0.4 cases/1,000py. Finally, incidence was increasing in landscapes “Cultivated forest” and “Young forest”. “Cultivated forest ≤10 km from Nyong” even presented an exponentially increasing incidence rate (R^2^ = 0.97 for exponential fit compared to R^2^ = 0.87 for linear fit).

## Discussion

Our study relied on the analysis of 787 BU cases over 125 months of follow-up, which to our knowledge is amongst the highest reported incidences in an endemic region with more than 10 years of continuous follow-up [28], [44]. We analysed BU spatio-temporal patterns and were able to reveal local-scale environmental determinants of BU incidence. We demonstrated that the Nyong River represented a major risk factor for BU, in conformity with previous studies of individual risk factors [30] and environmental MU detection [36], . We also identified different levels of risk along the river, which were associated to different environment profiles. We suggest that BU risk further increases with abundance of wetlands and with human modifications of landscape, such as cultivation and forest clearing. We also identified stable endemic areas and zones where incidence appears to be rising.

This work benefited from several methodological improvements compared to previous studies. By using the SPODT algorithm for identification of risk zones, we obtained a more accurate description than other studies [21], showing a decreasing risk gradient away from the Nyong River. By considering different categories of forest cover and management, cultivated and uncultivated wetlands, we accounted for local heterogeneities which would have been missed in broader analyses. Contrary to previous studies, which considered forest as a homogeneous category [21], [23], [28] and found it a risk factor, we used a detailed LULC classification, ground-truthed, and a small buffer radius (5 km instead of 20 km). We demonstrated that the different forest categories presented different risk levels according to their status regarding human activities, and that BU risk followed a dose-response relationship according to forest degradation [5], [46].

Our study showed that BU incidence spatio-temporal patterns are complex, but might be explained for a large part by landscape characteristics and heterogeneities. We identified the Nyong River as a major driver of BU incidence in the Akonolinga region, and local scale environmental variations in the landscapes along the river were associated to significantly different risk levels.

These variations, distinguishing between landscapes at high and intermediate BU-risk were principally the proportion of wetlands, and the type and extent of forest cover. The proportion of wetlands was evaluated topographically (% surface with TWI>18) or in LULC descriptions, where cultivated wetlands occupied a larger surface in high-risk landscapes (“Rural Nyong” and “Urban Nyong”) and forested wetlands in intermediate-risk landscapes (“Young forest” and “Cultivated forest”). The type and extent of forest cover reflected the level of human modifications. In the highest-risk landscapes, forest cover was reduced and corresponded mainly to cultivated young secondary forest. These landscapes, located in the densely populated part of the district, are shaped by intense agricultural pressure, as indicated also by the proportion of cultivated lands, including wetlands. The intermediate-risk landscapes near the Nyong River, “Cultivated forest <10 km from the Nyong River” and “Young forest”, were less modified by human activities and retained important forest covers. The observed increase in incidence during the study period could result from recent environmental modifications: using only a crude measurement, we showed that “Young forest” is one of the landscapes with the largest decrease in EVI, indicating a decrease in forest cover. These areas of increasing incidence are located downstream from the floodplain of the town of Akonolinga. Speculatively, MU could have spread along the Nyong colonising new environments.

The landscapes at lowest risk, “Forest 1”, “Forest 2” and “Cultivated forest >10 km from Nyong River”, were mainly composed of villages located far from the Nyong River and corresponding to the most preserved environments. In “Forest 1” landscape, BU incidence was about 100 times lower than in highest risk areas, while it was only about 50 times lower in “Forest 2” landscape. Even if not significant, we observed the same trend of BU risk increase with increasing forest degradation level.

We can propose that spatial variations of BU incidence in Akonolinga Health District resulted from the superimposition of two main factors: a high or low baseline risk related to the Nyong River proximity, and additional risks related to wetland abundance and environmental modifications by human activities. The role of the wetlands was supported by analyses of MU presence in Akonolinga water bodies, which showed that wetlands acted as a permanent reservoir of MU over the year, while other water bodies presented season-specific peaks of MU colonisation [45]. The increase in BU risk associated with human modifications of the environment could result from contact with newly accessible but pre-existing high-risk environments, from an increase in the number of contacts with risk sources due to populations increasing their activity range, or from the transformation of natural environments into high-risk sources by human activities (such as clearing wetlands for cultivation) [47]. The contribution of each phenomenon could be evaluated using chronological descriptions of the environment evolution, as well as of human practices.

The main limitation of this work was that it relied on “semi-active” case detection. We analysed data from cases which were diagnosed and treated at Akonolinga district hospital. They may not represent all the cases that occurred in the Akonolinga health district over the study period, since patients tend to seek traditional treatments as a first option [48]. However, since BU is a slowly progressing disease and difficult to cure even in hospitals, cases are likely to seek medical care at some point, eventually after failure of traditional treatment [48]. We analysed the spatial incidence trends over large periods of time, which probably allowed us to capture a large proportion of the incident cases over the study period, even patients with long delay to diagnosis. In addition, we cannot be sure that cases were infected in a given location, but given the activity patterns and our study scale, we can be confident that our work focuses on the main environments frequented by the populations.

This series of cases originated from a single treatment centre with a defined population-catchment area. The BU program in Akonolinga district has established a dynamic network of community correspondents in the villages, who contribute to population information and awareness, as well as case detection. Regular investigations are also performed in the area by medical staff and social workers in order to examine suspect cases and advocate for hospital treatment in a context where traditional treatment is generally the first option, despite free treatments [46]. This coverage ensured that a maximum number of incident cases were detected, diagnosed and treated, and therefore included in this analysis. Comparisons with previous data from cross-sectional surveys in Akonolinga region support our assumption that cases treated at Akonolinga hospital are representative of cases occurring in the Akonolinga district [17], [18] and support our description of localised increases in incidence.

Our results are consistent with previous results regarding individual risk factors. In a case-control study from 2006, wading in the Nyong swamps and not wearing long protective clothing while farming were identified as risk factors [30]. We showed that BU is associated to agricultural areas near the Nyong and suggested that the clearing and cultivation of swamps could have contributed to risk increase at the population level. This hypothesis was supported by interviews performed during an anthropologic study in Ekougou and Abem, two villages located near the Nyong flood plain in “Rural Nyong” landscape. The informants related BU to the practice of clearing swamps for vegetable cultivation, which started about 20–25 years ago in the flood plain near Akonolinga [37]. It would be interesting to document if these practices occur in landscapes with increasing incidence, and when they started. The informants also incriminated large bushfires in the 1980s, which deeply modified the ecosystem of the Nyong floodplain [37]. The increase in population could also explain a new need for land in more remote areas of the district, triggering deforestation and BU.

### Conclusion

The present work provides a quantitative assessment of the link between BU, slow flowing rivers, like the Nyong River, landscape features and their modifications by human activities. We clarify the role of forest, previously considered as a risk factor, by distinguishing pristine from human-perturbed ecosystems. We also underline major heterogeneities within Akonolinga endemic area, which presents stable high and low endemic zones, and zones with a rising incidence rate. Further studies regarding environment sampling for MU detection in endemic areas, or identification of risk-factors should take into account that environments at risk are defined at a very local scale. Surveillance of BU and active case search programs in endemic regions should also include the fact that BU geography can be substantially modified on a short time span, endangering new populations.

## Supporting Information

Checklist S1 **STROBE checklist.**
(DOC)

Text S1 **Supporting figures and detailed legends.** Includes: Figure S1: Space-time analysis of BU incidence in Akonolinga. Figure S2: Principal components analysis and selected results. Figure S3: Cumulative incidence graphs in 6 Akonolinga landscapes, 2002–2012.
(PDF)
