# Supplementary material for: Spatio-temporal Patterns and Landscape-Associated Risk of Buruli Ulcer in Akonolinga, Cameroon
Source: PLoS Negl Trop Dis. 2014 Sep 4;8(9):e3123. doi: 10.1371/journal.pntd.0003123 (PMC4154661; doi:10.1371/journal.pntd.0003123)
Supplement: Checklist S1 — STROBE checklist. (DOC) [file pntd.0003123.s001.doc]

STROBE Statement—Checklist of items that should be included in reports of ***cohort studies***

|  | Item No | Recommendation |
| --- | --- | --- |
| **Title and abstract** | 1 | (*a*) Indicate the study’s design with a commonly used term in the title or the abstract  ***This study relies on the analysis of a registry recording all patients presenting at a treatment centre from a defined region, but only aggregated analyses at the village level are performed. We do not think that it is relevant to include the term cohort or registry in the title of this article.*** |
|  |  | (*b*) Provide in the abstract an informative and balanced summary of what was done and what was found  ***OK*** |
| Introduction | | |
| Background/rationale | 2 | Explain the scientific background and rationale for the investigation being reported  ***See introduction section p3-4*** |
| Objectives | 3 | State specific objectives, including any prespecified hypotheses  ***See p5 lines 112-118*** |
| Methods | | |
| Study design | 4 | Present key elements of study design early in the paper  ***See p6, lines 128-137*** |
| Setting | 5 | Describe the setting, locations, and relevant dates, including periods of recruitment, exposure, follow-up, and data collection  ***See p6, lines 121-129*** |
| Participants | 6 | (*a*) Give the eligibility criteria, and the sources and methods of selection of participants. Describe methods of follow-up  ***See p6, lines 131-147*** |
|  |  | (*b*) For matched studies, give matching criteria and number of exposed and unexposed  ***NA*** |
| Variables | 7 | Clearly define all outcomes, exposures, predictors, potential confounders, and effect modifiers. Give diagnostic criteria, if applicable  ***Only diagnostic criteria apply, see p6, lines 140-144*** |
| Data sources/ measurement | 8* | For each variable of interest, give sources of data and details of methods of assessment (measurement). Describe comparability of assessment methods if there is more than one group  ***See p7, line 157 to p8, line 230*** |
| Bias | 9 | Describe any efforts to address potential sources of bias  ***Environment data: combination of many sources, see p8, line 191 to p9, line 230***  ***Statistical methods: diversity of approaches, see p9, line 258 to p12, line 296.*** |
| Study size | 10 | Explain how the study size was arrived at  ***See p6, lines 136-138*** |
| Quantitative variables | 11 | Explain how quantitative variables were handled in the analyses. If applicable, describe which groupings were chosen and why  ***OK*** |
| Statistical methods | 12 | (*a*) Describe all statistical methods, including those used to control for confounding  ***See p9, line 232 to p12, line 303*** |
|  |  | (*b*) Describe any methods used to examine subgroups and interactions  ***NA*** |
|  |  | (*c*) Explain how missing data were addressed  ***Case data: p6, line 137-138***  ***Environment data: p13, line 311-312*** |
|  |  | (*d*) If applicable, explain how loss to follow-up was addressed  ***NA*** |
|  |  | (*e*) Describe any sensitivity analyses  ***NA*** |
| Results | | |
| Participants | 13* | (a) Report numbers of individuals at each stage of study—eg numbers potentially eligible, examined for eligibility, confirmed eligible, included in the study, completing follow-up, and analysed  ***See p13, lines 306-315*** |
|  |  | (b) Give reasons for non-participation at each stage  ***See p13, lines 306-315*** |
|  |  | (c) Consider use of a flow diagram  ***Not necessary, only 3 steps.*** |
| Descriptive data | 14* | (a) Give characteristics of study participants (eg demographic, clinical, social) and information on exposures and potential confounders  ***Case data: all analyses are performed at the village level.***  ***Environment data: table 1 presents the characteristics of the village groups (group exposure categories)*** |
|  |  | (b) Indicate number of participants with missing data for each variable of interest  ***None*** |
|  |  | (c) Summarise follow-up time (eg, average and total amount): ***See p13, line 306*** |
| Outcome data | 15* | Report numbers of outcome events or summary measures over time  ***See p13, 318-320 and Figure 1A.*** |
| Main results | 16 | (*a*) Give unadjusted estimates and, if applicable, confounder-adjusted estimates and their precision (eg, 95% confidence interval). Make clear which confounders were adjusted for and why they were included  ***See table 2 and 3; and result section*** |
|  |  | (*b*) Report category boundaries when continuous variables were categorized  ***OK*** |
|  |  | (*c*) If relevant, consider translating estimates of relative risk into absolute risk for a meaningful time period  ***See p 16, 390-398, calculation of incidence rates for landscape groups with stable incidence rates*** |
| Other analyses | 17 | Report other analyses done—eg analyses of subgroups and interactions, and sensitivity analyses  ***All analyses done were reported.*** |
| Discussion | | |
| Key results | 18 | Summarise key results with reference to study objectives  ***See p17 lines 401-410*** |
| Limitations | 19 | Discuss limitations of the study, taking into account sources of potential bias or imprecision. Discuss both direction and magnitude of any potential bias  ***See p19-20 lines 467-488*** |
| Interpretation | 20 | Give a cautious overall interpretation of results considering objectives, limitations, multiplicity of analyses, results from similar studies, and other relevant evidence  ***See p18-19*** |
| Generalisability | 21 | Discuss the generalisability (external validity) of the study results  ***See p18, line 451 to p19, line 463; p20, lines 488-500 and conclusion.*** |
| Other information | | |
| Funding | 22 | Give the source of funding and the role of the funders for the present study and, if applicable, for the original study on which the present article is based  ***Done in the Funding section****.* |

*Give information separately for exposed and unexposed groups.

**Note:** An Explanation and Elaboration article discusses each checklist item and gives methodological background and published examples of transparent reporting. The STROBE checklist is best used in conjunction with this article (freely available on the Web sites of PLoS Medicine at http://www.plosmedicine.org/, Annals of Internal Medicine at http://www.annals.org/, and Epidemiology at http://www.epidem.com/). Information on the STROBE Initiative is available at http://www.strobe-statement.org.
