# Supplementary material for: Spatio-temporal Patterns and Landscape-Associated Risk of Buruli Ulcer in Akonolinga, Cameroon
Source: PLoS Negl Trop Dis. 2014 Sep 4;8(9):e3123. doi: 10.1371/journal.pntd.0003123 (PMC4154661; doi:10.1371/journal.pntd.0003123)
Supplement: Text S1 — Supporting figures and detailed legends. Includes: Figure S1: Space-time analysis of BU incidence in Akonolinga. Figure S2: Principal components analysis and selected results. Figure S3: Cumulative incidence graphs in 6 Akonolinga landscapes, 2002–2012. (PDF) [file pntd.0003123.s002.pdf]

Supplementary text S1: Supporting Figures & detailed legends

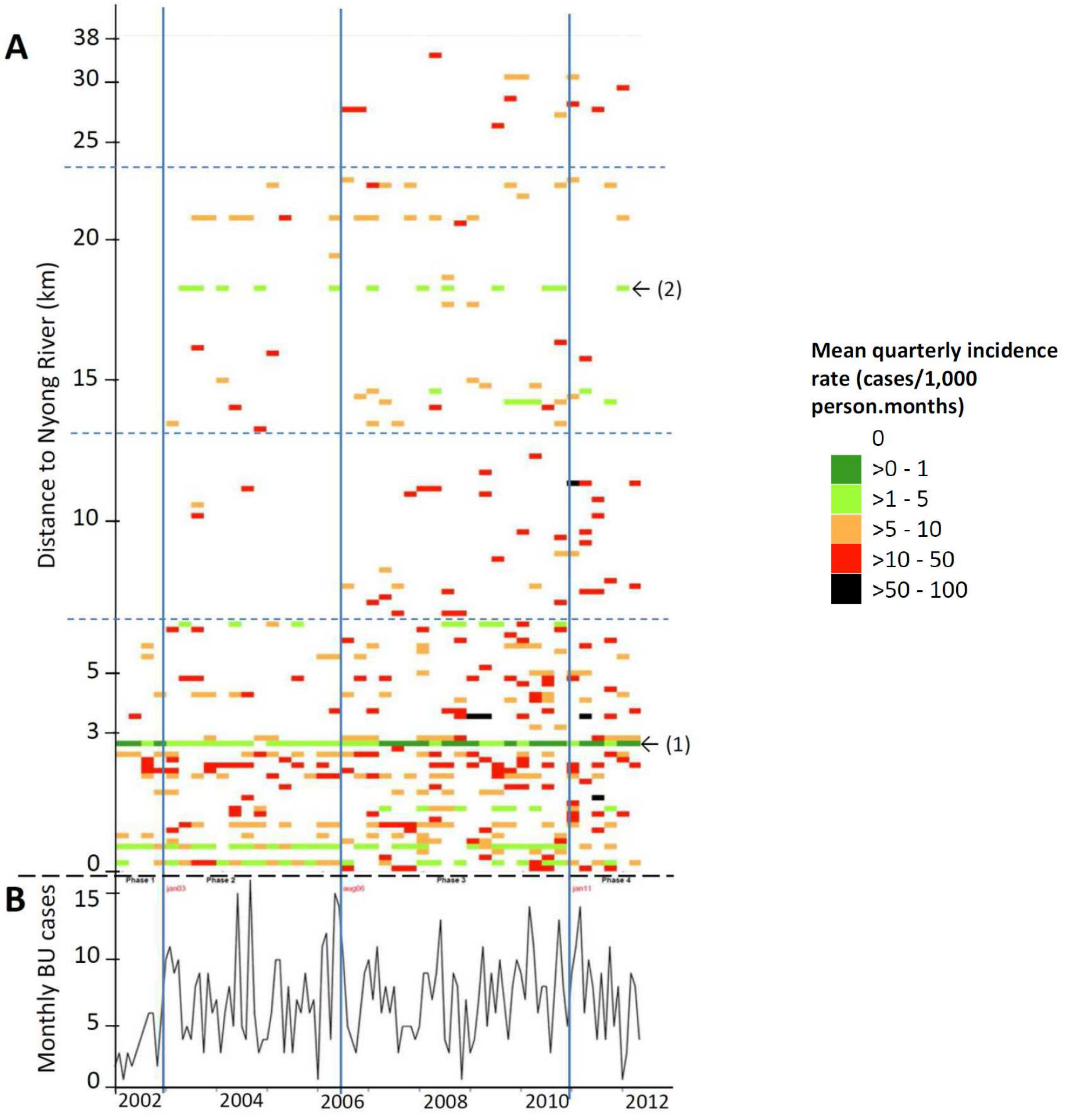

Figure S1: Space-time analysis of BU incidence in Akonolinga.

A: Identification of time-space patterns of BU incidence in Akonolinga on a heatmap of trimestrial BU incidence rate in the different villages of Akonolinga district 2002-2012.

Villages are ordered according to their distance to the Nyong, each village is represented by a horizontal bar of different colours. Each vertical bar represents a quarter year and the colour is proportional to the incidence. White corresponds to 0 incidence. The vertical solid lines indicate the different phases identified and horizontal dotted lines the different spatial zones.

Phase 1: The year 2002 corresponded to the beginning of the BU treatment program which was limited to Akonolinga town and its surroundings. From 2003, the program was covering the whole district (see S1B).
Phase 2: In the period from 2003-2006, villages within 0-7km from the river show incident cases. Sporadic cases also occurred in the 13-23km band.
Phase 3: From August 2006, an increase in incidence was observed in the villages located from 7 to 13km from the Nyong. Cases also started to be detected more than 23km away from the Nyong.
Phase 4: From January 2011 on, a decreasing trend was observed in the general incidence (see S1B).
(1) Akonolinga town (n=13,381 inhabitants), (2) Endom town (n=1,586)

B: Monthy time series of BU cases (raw data) in Akonolinga district from 2002 to 2012.

S2A

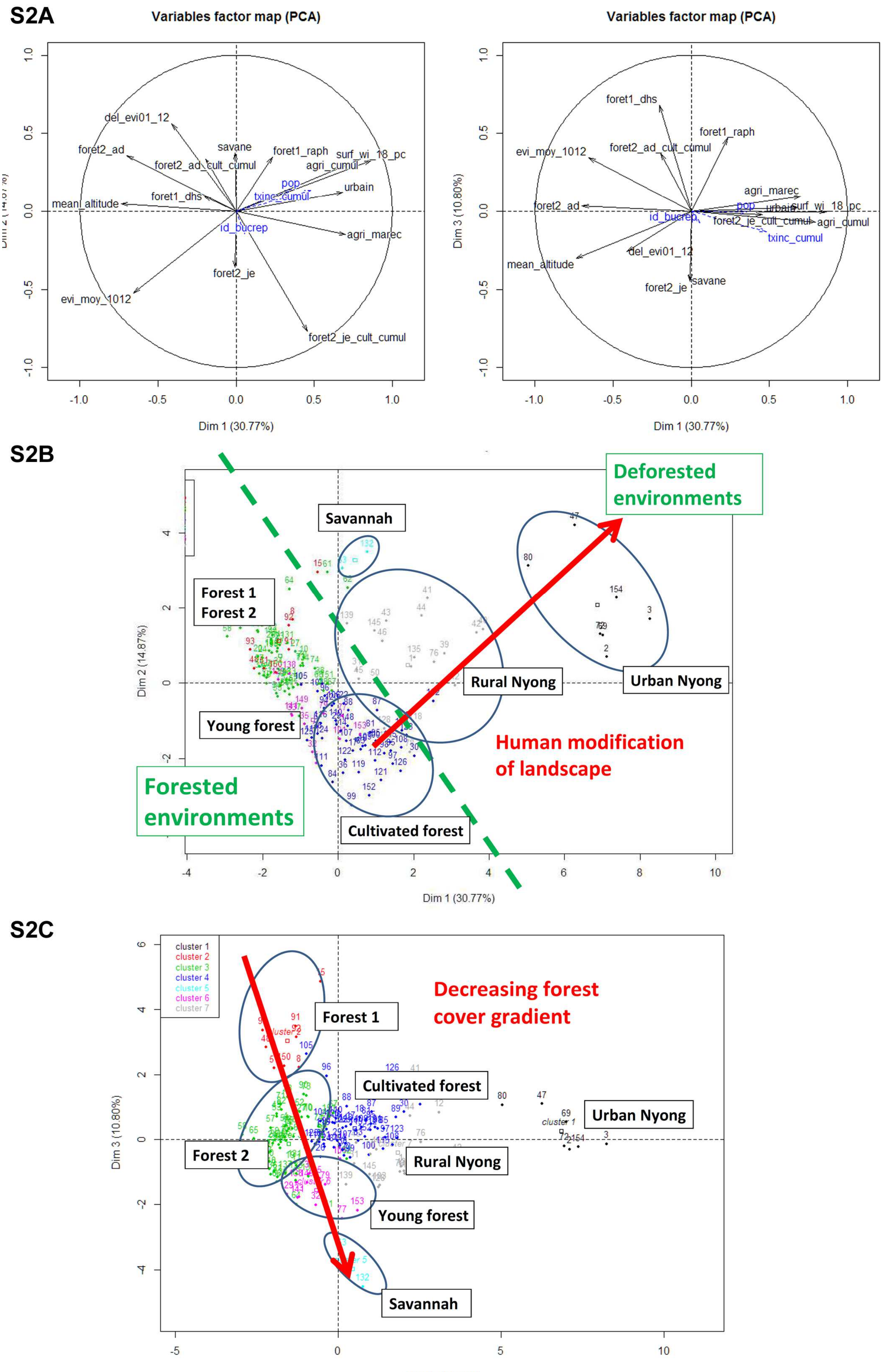

Variables factor map (PCA)
Dim 1 (30.77%)
Dim 3 (10.80%)
del_evi01_12
foret2_ad
savane
foret1_raph
surf_wi_18_pc
agri_cumul
foret2_ad_cult_cumul
pop
urbain
txinc_cumul
foret1_dhs
mean_altitude
id_bucrep
agri_marec
foret2_je
evi_moy_1012
foret2_je_cult_cumul

S2B

Deforested environments
Savannah
Forest 1
Forest 2
Rural Nyong
Urban Nyong
Young forest
Human modification of landscape
Forested environments
Cultivated forest
Dim 2 (14.87%)
Dim 1 (30.77%)

S2C

cluster 1
cluster 2
cluster 3
cluster 4
cluster 5
cluster 6
cluster 7
Decreasing forest cover gradient
Forest 1
Cultivated forest
Urban Nyong
Forest 2
Rural Nyong
Young forest
Savannah
Dim 3 (10.80%)
Dim 1 (30.77%)

Figure S2: Principal components analysis and selected results.

The following variables were introduced in the principal components analysis:

- Environment= % of buffer surface occupied by: urban, agriculture, wetlands, cultivated wetlands, savannah, primary forest (dense humid evergreen), forest with raffia trees, adult secondary forest, cultivated adult secondary forest, young secondary forest, cultivated young secondary forest
- Topography= altitude, % of buffer surface with a topographic wetness index >18 (wetland).
- Summary vegetation measurements= Mean EVI in December, Difference in EVI between dry season 2001-02 and 2011-2012.

S2A: Correlations circle, showing the contributions of the various variables to the 3 first principal components

Principal components analysis consists into obtaining new variables, the principal components, which contain the largest part of the information contained in the original variables, but summarised and combined with each other. On the circle, each original variable is represented as an arrow. Its direction indicates to which principal component it contributes most, and its length corresponds to the weight it contributed to the component.

For example, variable "urban" contributes positively to the first principal component (horizontal, directed to the right). "agri_marec", corresponding to cultivated wetlands proportion, also has a similar direction. On the contrary, "mean_altitude" contributes negatively to the first component. This indicates that, in Akonolinga district, villages with a high urban variable also have a high proportion of cultivated wetlands, but not a high altitude. These villages correspond to the "Akonolinga urban" landscape and are located on the right of figure S2B. The villages with a higher altitude, and also low urban and cultivated wetlands, are found on the left ("Forest 1" and "Forest 2"). Overall the two first components explain 45.64% of the total variance, and the first and third components 41.57 per cent of the total variance.

S2B: Representation of villages according to their coordinates on the first and second components coloured according to the landscape group.

S2C: Representation of villages according to their coordinates on the first and third components coloured according to the landscape group.

The representations in S2B and S2C show how villages were separated according to their environmental characteristics as indicated by the variables. We indicated the names of groups and the main features separating and grouping the villages: gradients, forest cover…

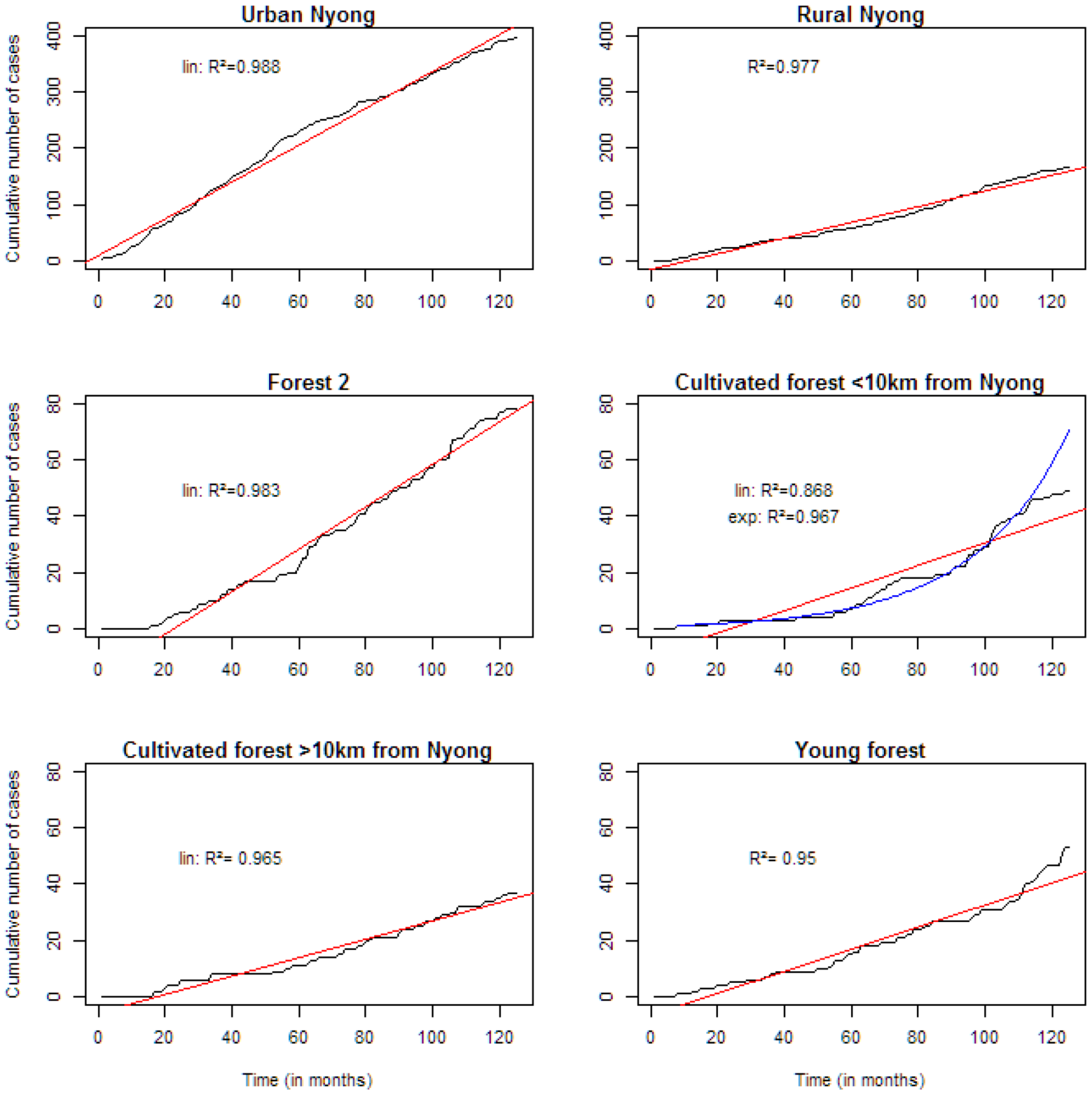

<u>Figure S3: Cumulative incidence graphs in 6 Akonolinga landscapes, 2002-2012.</u>

Linear fit is presented for each graph (with R^2 value). For landscape “Cultivated Forest, <10km from Nyong”, exponential and linear fits are presented with their respective R^2.

A good linear fit indicated that the monthly increase in case numbers was stable, and allowed us the calculation of an average incidence rate, over the period, within the landscape. When exponential curve fitted the data better we concluded that incidence was increasing.
